# Current Status of Obstetric Anaesthesia: Improving Satisfaction and Safety

**Published:** 2009-10

**Authors:** J Sudharma Ranasinghe, David Birnbach

**Affiliations:** 1 Professor, Department of Anesthesiology, Obstetric and Gynaecology and Public Health, University of Miami Miller School of Medicine

**Keywords:** Obstetric Anesthesia, Maternal mortality, Combined Spinal Epidural (CSE), Obesity, Preeclampsia, Hypotension

## Abstract

**Summary:**

The Centers for Disease Control and Prevention (CDC) reported in 2003 that although the maternal mortality rate has decreased by 99% since 1900, there has been no further decrease in the last two decades^1^. A more recent report indicates a rate of 11.8 per 100,000 live births^2^, although anaesthesia-related maternal mortality and morbidity has considerably decreased over the last few decades. Despite the growing complexity of problems and increasing challenges such as pre-existing maternal disease, obesity, and the increasing age of pregnant mothers, anaesthesia related maternal mortality is extremely rare in the developed world. The current safety has been achieved through changes in training, service, technical advances and multidisciplinary approach to care. The rates of general anaesthesia for cesarean delivery have decreased and neuraxial anaesthetics have become the most commonly used techniques. Neuraxial techniques are largely safe and effective, but potential complications, though rare, can be severe.

## Introduction

Although there are significant differences in maternal mortality rates between developed and developing countries, it appears that predisposing factors for anaesthesia related maternal mortality are similar, including inexperienced anaesthesia personnel, airway problems, and a lack of appropriate monitoring or resuscitation equipment. Maternal mortality can be decreased further by continuing to increase the use of neuraxial anesthesia and improving airway management skills because complications of regional anaesthesia may also involve airway management. Simulation is becoming an integral teaching methodology in anaesthesiology education, especially as relates to the management of crisis situations.

Factors such as advanced maternal age, black race, maternal obesity, cesarean delivery and multiple pregnancy (because of increased complications such as preeclampsia and peripartum hemorrhage) are known to increase the risk of maternal morbidity and mortality. In this review we will discuss the current status of anaesthesia for operative delivery, management of high risk parturient, and labor analgesia.

**Fig 1:**
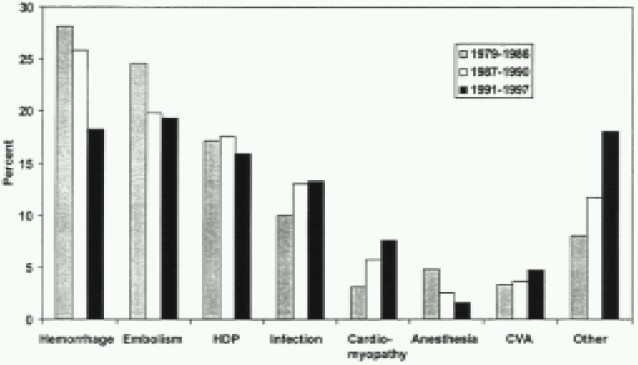
Cause-specific proportionate mortality for 1979-1986 , 1987-1990 and 1991-1997 , in the United States. HDP = hypertensive disorders of pregnancy;CVA = cerebrovascular accident.(Data from Berg CJ, Chang J, Callaghan WM, et al. Pregnancy-related mortality in the United States. 1991-1997. Obstet Gynecol. 2003;101:289-296 with permission).

Currently, the global maternal mortality rate (MMR) is approximately 400 per 100,000 live births, with significant inequality between developed and developing countries^3^. Despite recent advances, more than 99% of maternal deaths are occuring in the developing world. Most developed regions of the world now have MMR lower than 15^3^. More than half of maternal deaths are preventable and caused by hemorrhage, pregnancy-induced hypertension, infection, and ectopic pregnancy^2^. In a preliminary report, the anaesthesia-related maternal mortality was estimated at 1.3 per million live births in the United States.^4^ Table 1 reviews maternal mortality rates and Figure 1 reviews cause-specific proportionate mortality. Table 2 reviews causes of pregnancy-related deaths in the United States.

**Table 1:** Anaesthesia-Related Maternal Mortality Ratios in the United States and the United Kingdom, 1979-2005 (Rates reported refer to the risk of anaesthesia-related maternal deaths during pregnancy or up to 1 year after delivery per million live births in the United States or per million maternities in the United Kingdom)

**Triennium**	**United States (95% CI)**	**United Kingdom (95% CI)**
1979-81	4.3 (3.1-5.7)	8.7 (5.5-13.2)
1982-84	3.3 (2.3-4.5)	7.2 (4.3-11.4)
1985-87	2.3 (1.5-3.4)	2.6 (1.2-5.8)
1988-90	1.7 (1.1-2.7)	1.7 (0.7-4.4)
1991-93	1.4 (0.8-2.2)	3.5 (1.8-6.8)
1994-96	1.1 (0.6-1.9)	0.5 (0.1-2.6)
1997-99	1.2 (0.7-2.0)	1.4 (0.5-4.2)
2000-02	1.0 (0.5-1.7)	3.0 (1.4-6.6)
2003-05	Not available	2.8 (1.3-6.2)

(Data from Mhyre JM. Matenal Mortality.In Chestnut's Obstetric Anesthesia. 4^th^ edition. with permission.)

**Table 2:** Causes of Pregnancy-Related Death during Live Birth in the United States, 1991–1997

**Causes**	**Incidence**

Embolism	21.4%
Hypertensive disorders	19.4%
Hemorrhage	13.4%
Infection	12.6%
Cardiomyopathy	9.7%
CVA^*^	5.3%
Anesthesia	1.8%

(Data from Hawkins JL. Anesthesia-related Maternal Mortality. Clin Obstet Gynecol. 2003 Sep;46(3):679-87 with permission).

Anaesthesia related causes have now fallen to number seven on the compiled list of pregnancy related mortality^2^.

## Anaesthesia for Operative delivery

The rate of cesarean delivery in the United States has increased dramatically in the last three decades (4.5% in 1965 to 31.1% in 2006) and is the most common surgical procedure performed in the US^5^.As more patients become high risk, anaesthetic challenges also increase. Despite the increasing complexity including obesity, congenital heart disease and other chronic diseases, anaesthesia related maternal mortality has decreased over the past 50 years from 36 per 100,000 cesarean deliveries to one per 100,000 cesarean deliveries^6^. The current level of safety of cesarean delivery has been achieved via dynamic changes in the training and practice of obstetric anaesthesia. High-fidelity simulators have been developed to educate residents in anesthesiology. Simulation settings that mimic real life crisis situations in obstetric anaesthesia have been created by coupling mannequin with computer. These scenarios can effectively teach recognition and management of critical events with zero risk to patients, since if a mistake occurs it is to a mannequin.

Table 3 reviews case-fatality rates per million anaesthetics for cesarean delivery in the United States. The reduction in anaesthesia related maternal mortality and morbidity over recent decades can be largely ascribed to widespread use of neuraxial anaesthesia. Despite this increase, deaths associated with regional anaesthesia have declined markedly. The appreciation of cardiotoxicity of bupivacaine in the mid 1980s led to several changes in clinical practice to improve safety of neuraxial anaesthesia. These include withdrawal of 0.75% bupivacaine from obstetrics, widespread use of test doses, fractionation of epidural injection, and the use of dilute solutions by continuous infusion. In fact, there have been no reported deaths related to intravascular injection of local anaesthetics in a laboring patient for the last three decades. However, high block with delay in recognizing and treating its cardiorespiratory consequences, as well as lack of appropriate resuscitation equipment and drugs continue to result in maternal injury^7^.

**Table 3:** Case – Fatality Rates per Million Anesthetics for Cesarean Delivery in the United States

**Year of Death**	**GA (95% CI)**	**RA (95% CI)**	**Risk Ratio (95% CI)**
1979-1984	20.0 (17.7-22.7)	8.6 (7.8-9.4)	2.3 (1.9-2.9)
1985-1990	32.3 (25.9-49.3)	1.9 (1.8-2.0)	16.7 (12.9-21.8)
1991-1996	16.8 (8.9-28.7)	2.5 (1.2-4.5)	6.7 (3.0-14.9)
1997-2002	6.5 (2.1-15.3)	3.8 (2.3-6.1)	1.7 (0.6-4.6)

(Data from Mhyre JM. Matenal Mortality.In Chestnut's Obstetric Anesthesia. 4^th^ edition. with permission).

## General Anaesthesia

From 1979 to 1990, failed intubation was the leading cause of anaesthesia related maternal mortality^8^. According to the American Society of Anesthesiologists closed claim analysis^6^, there were seven difficult intubation injuries listed between 1991 and 1998, but none after 1999. Increased awareness, a steady decrease in the use of general anaesthesia for cesarean deliveries along with an increase in use of labor epidural analgesia, improved difficult airway protocols, and the rescue use of the laryngeal mask airway (LMA) have decreased the incidence and consequences of failed airway.

In the 1950s, in the United States, roughly 100 deaths per year were attributable to aspiration of gastric contents^9^. Currently, this is a very rare event due to increased awareness of the risk of aspiration due to pregnancy induced physiological changes, the implementation of fasting guidelines, adequate aspiration prophylaxis and increased utilization of regional anaesthesia. Since 1990, there have been only two closed claims of obstetric cases in the ASA database due to aspiration of gastric contents^7^. While some have recently recommended a more lenient approach to NPO status in labor, most obstetric anaesthesiologists continue to limit oral intake to clear fluids since any parturient may require an emergency cesarean delivery.

## Regional anaesthesia techniques

Currently, single-shot spinal anaesthesia with hyperbaric bupivacaine is frequently used for cesarean delivery. Most obstetric anaesthesiologists have discontinued the use of 5% lidocaine due to concerns about transient neurological symptoms. Spinal anaesthesia is simple to initiate, rapid in its effect and produces excellent operating conditions. However, continuous epidural analgesia can be titrated or topped up for prolonged surgery and may result in fewer abrupt haemodynamic fluctuations. Both techniques have a failure rate of 2—5%, even with experienced practitioners^10^. The introduction of combined spinal–epidural anaesthesia (CSEA) offers benefits of both techniques. CSEA also offers the prospect of reducing the anaesthetic failure rate of either technique used alone^11^.

## Anaesthesia in high risk parturient

## Obesity

Problems directly related to anaesthesia were found responsible for the death of six women in the UK according to the most recent CEMACH (Confidential Enquiries into Maternal and Child Health)^12^. Obesity was a factor in four of these women. Obesity is recognized as a pandemic nutritional disorder by the World Health Organization^13^. During the past 20 years there has been a dramatic increase in obesity in the USA and in 2004, 33.2% of women were found to be obese^14^. In obstetric practice, the challenges posed by obesity are enormous. The pathophysiological changes of obesity are multi-systemic and there is overall reduction in the functional reserve. The combination of obesity and pregnancy has a profound effect on the maternal cardiovascular system. Obesity is associated with a higher prevalence of hypertension, diabetes mellitus, and hyperlipidemia. It is also one of the risk factors for coronary artery disease and cerebrovascular accidents. Obese parturient also have an increased occurrence of preeclampsia, fetal macrosomia, and cesarean delivery rate^15^. There are also increased numbers of women who have had bariatric surgery in the US, posing new risks.

The incidence of difficult airway among obese parturients is much higher than among non-obese parturients^16^. Although neuraxial techniques can be very challenging, liberal use of these techniques administered early in labor is the most effective method for safe delivery of obese parturients. Continuous spinal catheter is often preferred in these patients because of the relatively high failure rate of epidural catheters and the importance of having a ‘working catheter’ in case an emergency operative delivery is required. Continuous spinal analgesia can readily be converted to surgical anaesthesia if necessary. This technique provides considerable predictability and reliability, allowing tight control of the anaesthetic level and duration of the block. Currently, the available catheters for this purpose are essentially epidural macro-catheters. Spinal microcatheters used in 1980s were subsequently withdrawn from the market due to concerns about cauda equine syndrome. The spinal catheter should be clearly labeled and all personnel should be made aware of the nature of the catheter to prevent accidental injection of medication doses intended for epidural use. One possible complication of spinal macrocatheters is postdural puncture headache (PDPH). However, it has been suggested that the risk of PDPH is significantly decreased in morbidly obese patients^17^. Several techniques have been described to reduce the incidence of PDPH following spinal catheters. These include, puncturing the dura with the bevel of the needle parallel to the longitudinal axis of the back, and leaving the spinal catheter in-situ for 2-24h^18^.

Ultrasound is an emerging technology now being utilized for neuraxial needle placement. It has the potential to identify the midline through visualization of the spinous process in the obese patient. Ultrasound can determine the optimal insertion point and the distance from the skin to the ligamentum flavum. It can also identify the exact interspace of needle placement, helping to prevent direct spinal cord trauma. In theASA closed claim analysis, there were 2 cases of spinal cord injury due to direct injection into the cord^7^. A poor agreement (up to 50% of cases) between palpation and ultrasound estimation of the specific lumbar interspace by anaesthesiologists has been reported^19^.

## Preeclampsia

Neuraxial techniques are considered a safe method of providing anaesthesia for the patient with preeclampsia and severe preeclampsia. This is due to avoidance of the risks associated with general anaesthesia such as exacerbated hypertension, failed intubation, and aspiration. Preemptive epidural catheter placement should be considered before temporal worsening of the condition and potential development of thrombocytopenia that might preclude a neuraxial block.

The avoidance of spinal anaesthesia for severe preeclamptics due to fear of abrupt hypotension has been questioned. Recent studies have shown that the incidence of hypotension during spinal anaesthesia for cesarean delivery is infrequent in preeclamptic patients^20^ and many anaesthesiologists are now using this technique.

## Preterm cesarean delivery

**Fig 2:**
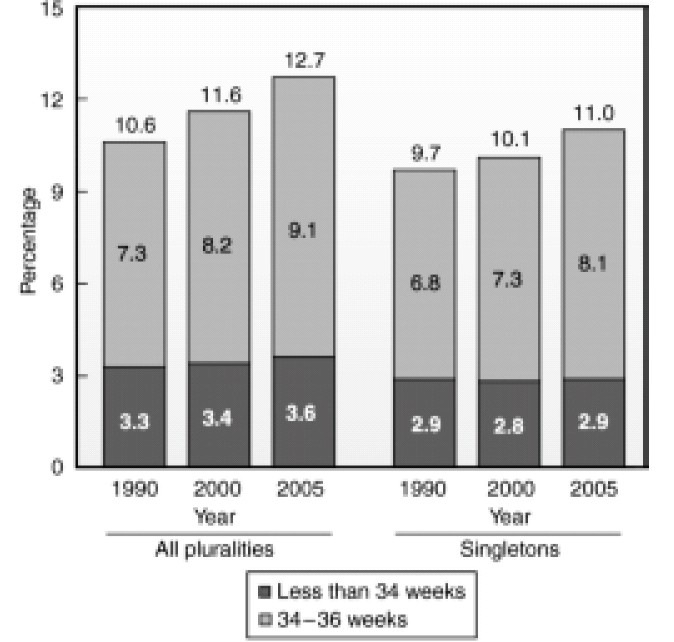
Preterm birth rates for all births and for singletons only: United States, 1990, 2000, and 2005. (Data From Martin JA, Hamilton BE, Sutton PD, et al. Births: Final data for 2005. Natl Vital Stat Rep 2007; 56:1-103.)

According to the US birth statistics there is increase in the preterm delivery rate^21^ with a 30% increase since 1981. This has been associated with a significant increase in cesarean delivery rates^22^ and requirement of anaesthesia services for the delivery. Fig 2 highlights the preterm birth rates in the United States since 1990.

Although commonly used, the main disadvantage of spinal anaesthesia is the risk of maternal hypotension with possible compromise of uteroplacental blood flow leading to fetal acidosis. In a large, retrospective, secondary analysis of EPIPAGE study of very preterm infants (VPIs of 27 to 32 weeks), Laudenbach et al^23^, reports a significantly higher risk of mortality in neonates when mothers received spinal anesthesia for cesarean, as compared to general or epidural anaesthesia. Neonatal mortality was 10.1% with general anaesthesia, 12.2% with spinal anaesthesia and 7.7% with epidural anaesthesia (adjusted odd ratio, 1.7; 95% confidence interval 1.1 to 2.6). The exact mechanism of this finding is not clear, but severe or sustained hypotension and excessive use of ephedrine^24^ (see below) were suggested as possible contributing factors. The authors suggest ‘VPIs may be very susceptible to uteroplacental hypoperfusion, an event that may be missed because of apparent stable hemodynamics in the mother’.

## Prevention of hypotension during neuraxial anaesthesia for cesarean delivery:

Hypotension is a common clinical problem following neuraxial blockade and is associated with morbidity for both mother (nausea, vomiting) and fetus (acidosis). Techniques used to prevent hypotension include intravenous fluids and sympathomimetic drugs.

## Intravenous fluids:

Based on current evidence, intravenous prehydration has poor efficacy, probably because of rapid distribution^25^. It has been shown that administration of a fluid bolus starting at the time of injection of neuraxial anaesthetic (cohydration) is more effective because maximum effect can be achieved at the time of the block and consequent vasodilatation^26^.

## Sympathomimetics : Ephedrine versus phenylephrine

Sympathomimetics with more alpha adrenergic activity have been avoided in obstetric practice in the past due to the fear of uterine artery vasoconstriction with resulting compromise in uterine blood flow. However, a recent study^24^ has demonstrated that ephedrine is in fact, associated with a greater propensity toward fetal acidosis compared with phenylephrine. Ephedrine was shown to cross the placenta to a greater extent and undergo less early metabolism and/or redistribution in the fetus compared to phenylephrine. Therefore, fetal acidosis is probably related to metabolic effects secondary to stimulation of fetal beta adrenergic receptors. This latest finding cautions against the use of large doses of ephedrine in the treatment of maternal hypotension; however, the current practice is to use ephedrine or neosynephrine depending on the maternal heart rate.

## Supplemental oxygen during cesarean delivery

It is common practice to provide supplementary oxygen to the mother during neuraxial anaesthesia for cesarean delivery. In recent years possible neonatal harm from high maternal oxygen levels has raised concerns. Free radicals liberated from high oxygen levels attack the lipid membrane (lipid peroxidation) and may cause tissue damage. However, studies have revealed not only that high maternal oxygen did not increase lipid peroxidation in the fetus (i.e., not harmful), but also maternal oxygen did not result in better fetal oxygenation or acid-base status even when the uterine-incision to delivery time was prolonged^27^. Based on this, we believe that it is reasonable to continue supplemental oxygen during emergency cesarean but consider it unnecessary during elective cesarean in healthy patients.

## Postoperative pain management:

When neuraxial anaesthesia is utilized for cesarean delivery, neuraxial morphine is currently the leading agent utilized for post-cesarean analgesia due to its long duration of action, low cost, and pain relief in the absence of motor or sympathetic blockade facilitating early ambulation.

## Obstetric Hemorrhage

Despite the ready availability of blood and blood products, hemorrhage remains a leading cause of maternal death in the United States. Evidence suggests that the incidence of placenta accreta is rising primarily due to increase in cesarean delivery rate, thus increasing the risk of hemorrhage^28^.

Prophylactic insertion of internal iliac artery balloon occlusion catheters (IIAOBC) via the femoral arteries appears to be a safe and effective method in controlling anticipated life-threatening bleeding in patients with placenta accreta. In patients with persistent post partum hemorrhage (PPH) following cesarean delivery, arterial embolization can be used to control bleeding as well as to reduce the need for hysterectomy, preserving future fertility. The major complications that have been reported from pelvic arterial embolization include thrombosis of the left popliteal artery, ischemia of sciatic nerve, necrosis of the cervix and proximal vaginal epithelium (with subsequent full recovery) and hematoma in the groin^29^. The long term effects of pelvic arterial embolization have not been thoroughly investigated. Therefore, when making a decision to use prophylactic IIAOBC, the risk of complications should be weighed against the benefits of hemorrhage control and preservation of fertility.

## Recombinant VIIa (rFVIIa):

Recombinant FVIIa is a rapidly acting drug licensed for the treatment of bleeding episodes in patients with hemophilia. Although PPH is an ‘off label’ indication, many centers are now using rFVIIa immediately before the decision to perform a hysterectomy, especially when arterial embolization is not available. The European Registry suggests 80-90% effectiveness in obstetric bleeding^30^. The drug is extremely expensive (> $10,000 a dose) and has thrombogenic potential. A recent study reported that the fibrinogen level can be used to guide the management of PPH. When the fibrinogen level is below 2 g L^-^ 1 there is a high risk of bleeding^31^. However, the cornerstone of the management of massive PPH remains a combination of surgery and/or medical management with conventional blood products and uterotonic drugs.

## Erythrocyte salvage during cesarean delivery:

There are two substantial difficulties in assessing the relative safety of intraoperative erythrocyte salvage during cesarean section^32^.

1. The pathophysiologic etiology of amniotic fluid embolism is not clear. Therefore studies cannot evaluate whether processing salvaged blood reduces the causative agent (even with leukodepletion filter).
2. The incidence of amniotic fluid embolism is low, approximately 1/8,000 to 1/80,000. Therefore, large prospective randomized studies are necessary to document the safety of cell salvage technique during cesarean delivery.

Therefore, some authors believe the cell salvage should be limited to those times when there is no alternative to augment oxygen carrying capacity^32^. However, the risk benefit ratio seems to have changed in favor of cell salvage in recent years^33^. Several bodies (CEMACH, ACOG and the OAA/AAGBI) have endorsed the use of cell saver in obstetric hemorrhage, even if patient accepts allogenic blood.

## Labor analgesia

Epidural analgesia has been used to alleviate labor pain for almost 50 years. Currently, the most commonly used technique is continuous epidural infusion, which avoids problems associated with intermittent bolus techniques such as uneven analgesia and possible increased infection rate. Bupivacaine has been the standard local anaesthetic for labor for many years. Recently, ropivacaine has been compared to bupivacaine for this purpose and shown to provide very satisfactory labor analgesia with a possible reduced incidence of motor blockade and decreased cardiotoxicity. The new local anaesthetics are however, considerably more expensive than bupivacaine. With the widespread use of ultra-dilute epidural infusions of bupivacaine and CSE technique motor blockade is uncommon and, it is very unlikely that systemic toxicity will be a problem during labor epidural analgesia. Therefore there seems to be no clinical justification to routinely use more expensive local anesthetics such as ropivacaine in the current labor analgesia practice.

The management of epidural analgesia during labor has changed over the past two decades. The addition of neuraxial opioids (such as fentanyl) to local anaesthetics allow adequate labor analgesia with very dilute solutions of local anaesthetics, thus minimizing potential adverse effects on the progress of labor and lower extremity motor block. Low dose local anaesthesia may allow women ambulate while in labor (termed in the laypress “the walking epidural”). Although not yet in clinical practice, recent studies have demonstrated effectiveness of clonidine and neostigmine as adjuncts to local anesthesia for labor analgesia^34^,^35^.

In recent years, the CSE (combined spinal epidural) technique has gained popularity in obstetric practice to provide optimal analgesia for parturients because it offers the possibility of combining the rapid onset of subarachnoid analgesia with the flexibility of continuous epidural analgesia. The duration of spinal analgesia is between 2 and 3 h, depending on which agent or agents are chosen. The original description of spinal analgesia involved sufentanil or fentanyl, but the addition of isobaric bupivacaine to the opioid produces greater density of sensory blockade with longer duration while still minimizing motor blockade^36^. Originally, 25 mcg of fentanyl or 10 mcg of sufentanil with 2.5 mg of bupivacaine was advocated, but more recent studies have suggested using smaller doses of opioid combined with smaller doses of local anaesthetic^37^. Many clinicians now routinely use 5 mcg of sufentanil or 15 mcg of fentanyl with 1.25 mg of bupivacaine intrathecally. Fentanyl is widely used as an intrathecal agent for labor analgesia because of its low cost, rapid onset and profound analgesia without motor blockade. Intrathecal fentanyl creates a ‘seamless’ transition from spinal to epidural by providing adequate duration of analgesia for an epidural infusion started immediately after intrathecal injection. Serious maternal side effects of intrathecal fenanyl are infrequent. Lipophilic opioids however, may cause early-onset respiratory depressiontypically within 30 min. The ASA Task Force recommends that after neuraxial lipophilic opioids continual respiratory monitoring should be performed for a minimum of 2 h after bolus administration or discontinuation of the infusion^38^.

When compared with conventional epidural analgesia for labor, the incidence of overall failure was shown to be significantly lower in patients receiving CSE analgesia. This difference may be due to the option to confirm questionable epidural needle location by successful spinal injection^39^.

Tsen et al reported that CSE analgesia, when administered to nulliparous parturients in early labor, resulted in significantly more rapid cervical dilatation compared with standard epidural analgesia^40^. This is probably due to the rapid reduction in maternal catecholamines secondary to immediate pain relief with CSE analgesia and has been confirmed in a more recent study by Wong et al^41^.

One concern with the CSE technique is that the function of the epidural catheter is uncertain until after the spinal analgesia has receded. This can be worrisome if an emergency procedure is required prior to this time period. However, epidural catheters inserted via CSE technique have been demonstrated to have a higher probability of being in the epidural space as compared to catheters inserted in the stand-alone epidural technique^42^

Many studies have evaluated CSE versus conventional epidural analgesia technique. They found no difference in obstetric or neonatal outcome due to the choice of anaesthetic technique. There was no increased incidence of positional headache due to intentional dural puncture in the CSE group using a 27 gauge (pencil point) spinal needle^43^.

Recently, patient controlled epidural analgesia (PCEA) techniques have evolved to provide more flexible analgesia. PCEA allows analgesia to be tailored to the individual needs of the parturient throughout the different phases of labor. The administration of a modest proportion (33%) of the maximum hourly demand dose as a background infusion reduces the need for physician-administered supplementation. This can be helpful in a busy obstetric unit^44^. Computer integrated systems have been examined to provide seamless analgesia from induction of neuraxial block to delivery^45^.

A study by Sebastian et al showed that employing a regimen of regularly scheduled automated intermittent boluses could improve the analgesic function of the epidural catheter^46^. It has been shown in experiments that the spread of an infusate from a multi-orificed catheter is more extensive if regular boluses were used instead of a continuous infusion. This was shown to be true despite a similar rate of discharge. In future, infusion devices may become available to allow programmed boluses^46^.

Theoretically, CSE can be associated with an increased risk of meningitis compared with straight epidural; this is as a result of puncture of the protective dural barrier and subsequent adjacent placement of a foreign body-the epidural catheter. Beginning in the mid 1990s, several case reports of meningitis following CSE have appeared in the journals^47^. Maximal sterile barrier precautions should always be followed during neuraxial procedures. The current advice is the removal of all jewelry (rings, watches) and washing hands with a disinfectant agent before wearing sterile gloves prior to the block. This agent can be either conventional antiseptic –containing soap and water or waterless alcohol-based gels or foams. The use of gloves does not obviate the need for hand hygiene. There is no current recommendation on wearing sterile gowns. However, caps and face masks should be worn for all neuraxial techniques. Chlorhexidine antiseptic for skin sterilization has been shown to have a faster onset of action with longer duration. However, the US Food and Drug Administration has not approved chlorhexidine for neuraxial use and there is an animal study demonstrating potential neurotoxicity.^48^

The effect of epidural analgesia on labor outcomes has been a controversial topic for many years. A recent article by Wong et al^41^ concluded:

1. Neuraxial analgesia with low concentration epidural infusions (bupivacaine 0.0625% with 2 micg / ml of fenatnyl or 0.125% bupivacaine) does not increase the risk of cesarean delivery or instrumental vaginal delivery.
2. Neuraxial analgesia in early labor (< 4 cm dilatation) does not increase the rate of cesarean delivery**.** Compared to systemic analgesia, it provides better analgesia and shorter duration of labor.

## Intravenous opioid analgesia with Remifentanil:

Remifentanil has been used successfully to manage labor pain in patients who present with contraindications to epidural analgesia. Recent comparative studies show that intravenous patient controlled (IV-PCA) remifentanil provides better analgesia than IV-PCA meperidine^49^

Remifentanil is a relatively new synthetic ultrashort-acting, rapid onset (approximately 1 minute) µ -receptor agonist. It has a constant context-sensitive halftime of approximately 3 min. Remifentanil crosses the placenta readily, but is rapidly metabolized and/or redistributed by the fetus.

The Healthy People 2010 target for maternal mortality is 3.3 per 100,000 live births^2^. Clearly, anaesthesiarelated maternal mortality rates are decreasing, but there is still room for improvement. In many obstetric disasters, early intervention and skills of an anaesthesiologist can make the difference between life and death. Effective communication and good team work between anaesthesiologist and obstetrician is essential. Future developments in obstetric care should focus on multidisciplinaryapproach utilizing the skills and knowledge of all those involved in the care of the pregnant patient.
